# Combined TP53 status in tumor-free resection margins and circulating microRNA profiling predicts the risk of locoregional recurrence in head and neck cancer

**DOI:** 10.1186/s40364-024-00576-y

**Published:** 2024-03-05

**Authors:** Federica Ganci, Matteo Allegretti, Carlotta Frascolla, Francesca Spinella, Francesca Rollo, Andrea Sacconi, Pascale De Valentina, Alina Catalina Palcau, Valentina Manciocco, Mariavittoria Vescovo, Ettore Cotroneo, Francesca Blandino, Maria Benevolo, Renato Covello, Paola Muti, Sabrina Strano, Antonello Vidiri, Giulia Fontemaggi, Raul Pellini, Giovanni Blandino

**Affiliations:** 1 Translational Oncologic Research Unit, IRCCS Regina Elena National Cancer Institute, Via Elio Chianesi 53, 00144 Rome, Italy; 2 Department of Research and Development, Eurofins Genoma Group, Rome, Italy; 3 Clinical and Technical Department Management, Eurofins Genoma Group, Rome, Italy; 4 Pathology, IRCCS Regina Elena National Cancer Institute, Via Elio Chianesi 53, 00144 Rome, Italy; 5 Otolaryngology-Head and Neck Surgery, IRCCS Regina Elena National Cancer Institute, Via Elio Chianesi 53, 00144 Rome, Italy; 6 Department of Health Research Methods, Evidence, and Impact, Faculty of Health Sciences, McMaster University, Hamilton, ON Canada; 7 Department of Biomedical, Surgical and Dental Sciences, University of Milan, Milan, Italy; 8 SAFU Unit, IRCCS Regina Elena National Cancer Institute, Via Elio Chianesi 53, 00144 Rome, Italy; 9 Radiology and Diagnostic Imaging, IRCCS Regina Elena National Cancer Institute, Via Elio Chianesi 53, 00144 Rome, Italy

**Keywords:** HNSCC, Resection margins, Local recurrence, TP53, microRNA profiling, Liquid biopsy

## Abstract

Locoregional recurrences represent a frequently unexpected problem in head and neck squamous cell carcinoma (HNSCC). Relapse often (10–30%) occurs in patients with histologically negative resection margins (RMs), probably due to residual tumor cells or hidden pre-cancerous lesions in normal mucosa, both missed by histopathological examination. Therefore, definition of a ‘clean’ or tumor-negative RM is controversial, demanding for novel approaches to be accurately explored. Here, we evaluated next generation sequencing (NGS) and digital PCR (dPCR) as tools to profile TP53 mutational status and circulating microRNA expression aiming at scoring the locoregional risk of recurrence by means of molecular analyses. Serial monitoring of these biomarkers allowed identifying patients at high risk, laying the ground for accurate tracking of disease evolution and potential intensification of post-operative treatments. Additionally, our pipeline demonstrated its applicability into the clinical routine, being cost-effective and feasible in terms of patient sampling, holding promise to accurately (re)-stage RMs in the era of precision medicine.

**Supplementary Information:**

The online version contains supplementary material available at 10.1186/s40364-024-00576-y.

## To the editor

**Fig. 1:**
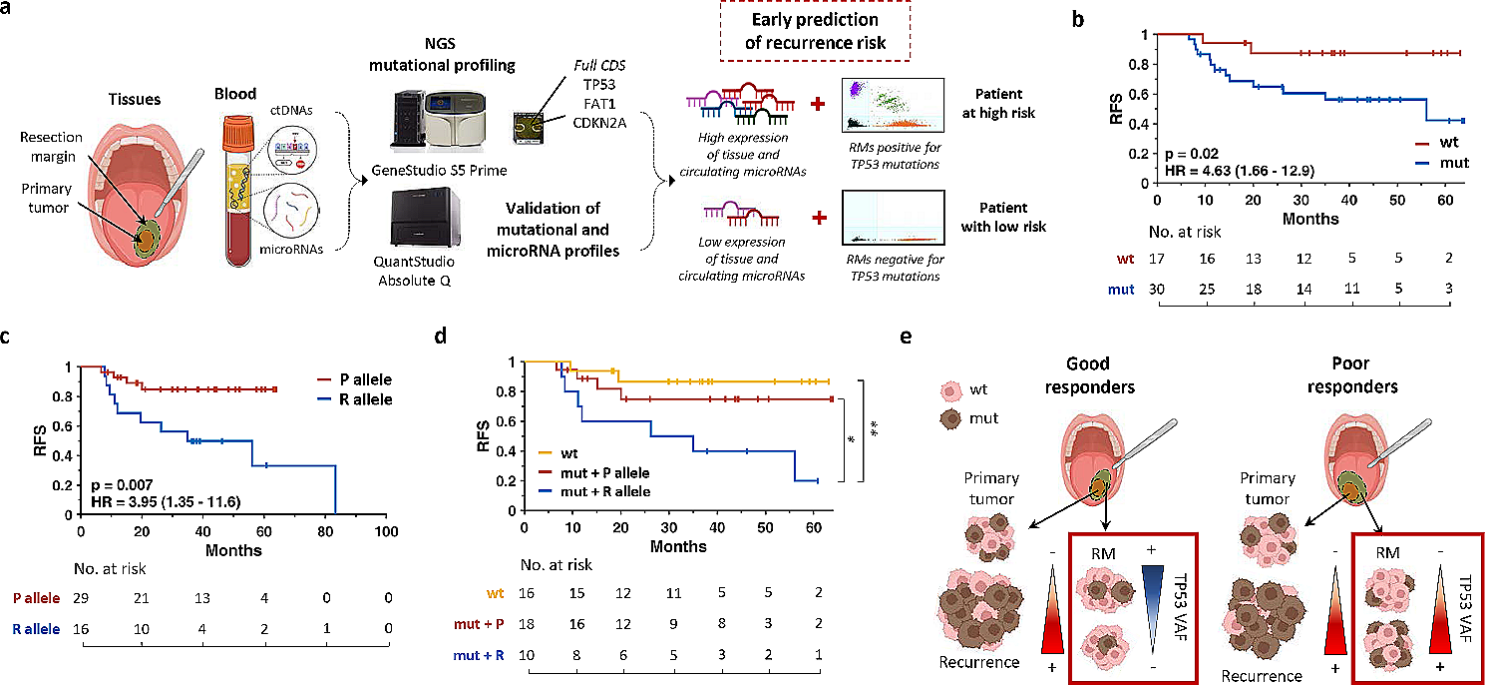
Molecular profiling of RMs and patient outcome. (a) Model of liquid biopsy (LB) and tissues analyses for early prediction of recurrence. TP53 mutational status and expression of the prognostic microRNAs signature have been assessed in resection margins (RMs) and LB samples from representative HNSCC patients by NGS, qPCR and dPCR. The combination of TP53 status and microRNAs expression in histologically tumor-free RMs and in sera samples taken at different time points (i.e., before and after surgery) may early predict tumor persistence or the risk of local recurrence in HNSCC. (b-c) Kaplan-Meier (KM) analyses of RMs according to (b) TP53 mutational status (wt or mutated, red or blue, respectively) or (c) the TP53 p.P72R polymorphism (P or R allele, red or blue, respectively). P72-positive RMs includes patients with P allele VAF > 75% while R72-positive RMs contains samples harboring heterozygous P/R or homozygous R alleles. CI values (95%) are shown within parenthesis. (d) Merged KM analyses resulting from TP53 mutational status and P72R polymorphism. (e) Representative model of TP53 abundance (VAF, brown cells) dynamics in tumor and resection margins. CDS: coding sequence; HR: hazard ratio; RFS: recurrence-free survival; VAF: variant allele frequency; wt: wild type; mut = mutated. *: p = 0.06; **: p = 0.006

**Fig. 2:**
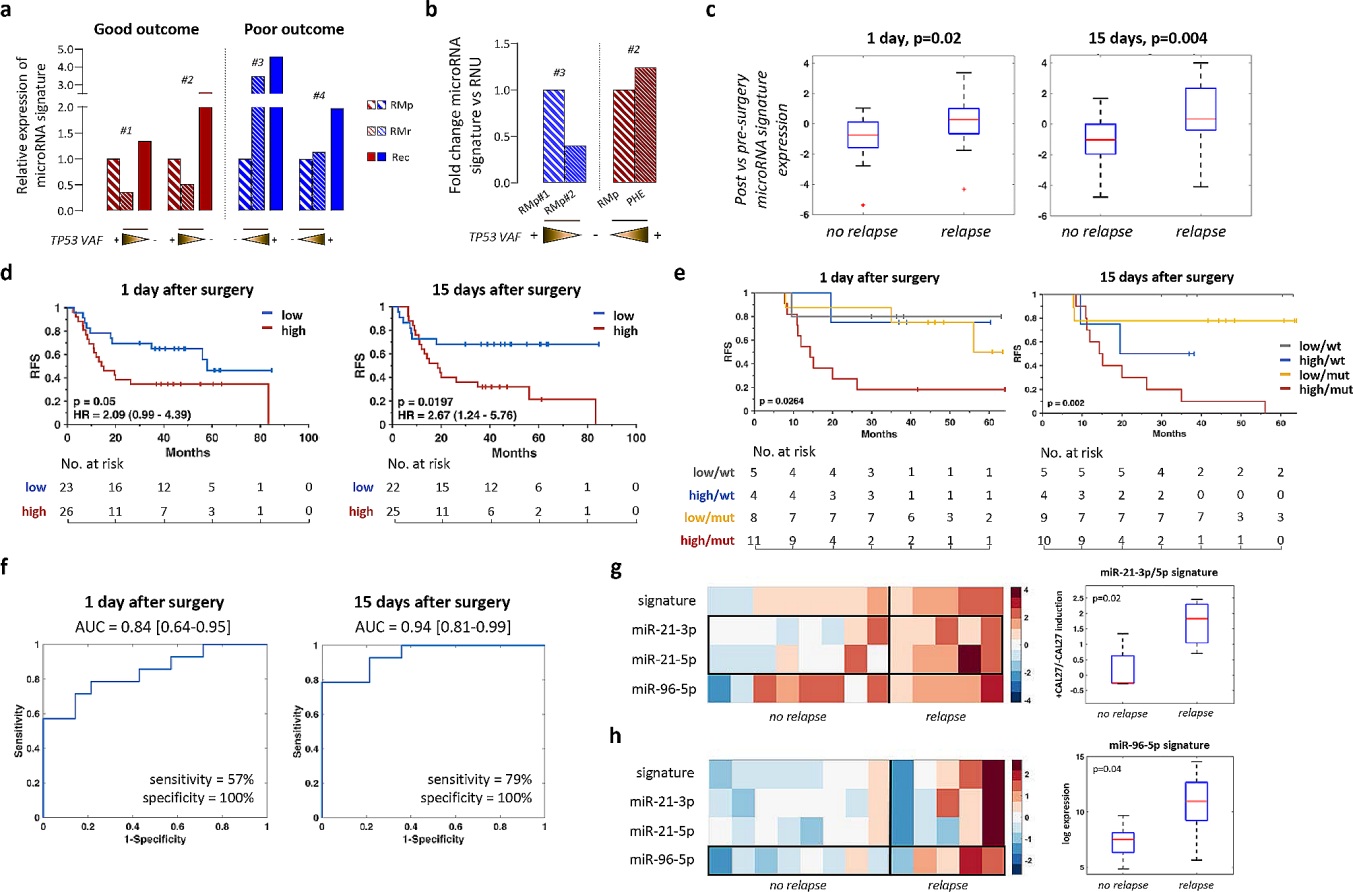
Prognostic value of circulating microRNAs and TP53 mutational status of matched RMs. (a) A 4-prognostic microRNA signature was assessed on RMs collected at disease onset (RMp) or relapse (RMr) and compared with tumor relapse. Patients were stratified according to their clinical outcome. Relative expression of microRNAs is shown. Dynamics of TP53 VAF is indicated. (b) The same microRNA signature was assessed on tissues from pt#3 (left) and pt#2 (right). For pt#3, the RM of the first primitive tumor (RMp#1) developed on the palatine tonsil, on which he recurred, was compared with the RM of the second primitive tumor (RMp#2) on floor of the mouth, on which the patient has ever not recurred. For pt#2, RMp has been compared with the PHE lesion, developed one year before the first recurrence. Relative expression of microRNAs is shown. Dynamics of TP53 VAF is indicated. (c-d) Box plot and KM analysis showing the diagnostic and prognostic value of our circulating microRNA signature to early predict local recurrence. microRNAs expression of sera collected at 1 day or 15 days post-surgery has been normalized to microRNAs expression of matched pre-surgery sera. (e-f) KM and ROC curve analyses according to mutational status of RMs and microRNAs signature expression at 1 day (left) or 15 days post-surgery (right). For each KM, HR value and the relative confident interval (CI) 95% has been indicated. (g) Supervised clustering (left) analysis representing the expression of the 3 prognostic microRNAs in normal tissues from 13 HNSCC patients cultured with CM from Cal27 cells according to patient’s outcome. Colors represent folds of modulation of CM vs RPMI. Box plot (right) representing the expression level of miR-21-3p and miR-21-5p significantly (p=0.02) up-regulated in histologically tumor-free tissues from n=5 recurrent patients vs n=8 patients with no evidence of disease (NED) for at least 36 months cultured with CM as compared to the same tissues cultured with RPMI. (h) Supervised clustering (left) analysis representing the expression of the 3 prognostic microRNAs in normal tissues from 13 HNSCC patients cultured with RPMI medium according to their clinical outcome. Box plot (right) showing miR-96-5p up-regulation (p=0.04) in histologically tumor-free tissues from n=5 recurrent patients vs n=8 patients with NED for at least 36 months, cultured in the presence of RPMI. Raw data of miRNAs expression and the relative patient outcome are available in Suppl. Tables, sheet 5-6. HR: hazard ratio; NED: no evidence of the disease; Rec: recurrence; RFS: recurrence-free survival.

Elucidation of HNSCC genomic landscape has provided novel insights for this neoplasia, laying the ground for multimodal approaches. Therapeutic efficacy, however, still faces with locoregional recurrences, which frequently represent an unexpected problem. Relapse often originates in patients with histologically negative resection margins (RMs) [1], thus demanding for combined approaches assessing cancer-associated alterations (Fig. 1a). By performing the mutational profiling of the 3 most frequently mutated genes in HNSCC [2–4] in paired RMs and tumors from 47 HPV-negative patients (Group 1, see Suppl. Figure 1a and Suppl. Tables, sheet 2), we proved that 64% of cases had alterations in RMs (Suppl. Tables, sheet 4), almost all affecting *TP53*. Independently from the overlap with primitives, patients with RMs carrying TP53mutants showed a significantly higher probability to develop local recurrence (Fig. 1b). Additionally, when incidence of TP53 p.P72R single nucleotide polymorphism (SNP; rs1042522) (Fig. 1c), previously linked to both reduced clinical outcome and therapeutic response due to its role in affecting TP53 interactions with coactivators [5–7] was taken into account, its integration further increases the prognostic value of molecular profiling (Fig. 1d). To corroborate the impact of RMs status, tissues from 4 recurrent patients with different clinical behaviors (e.g., good vs. poor outcome, Suppl. Figures 2–5) were deeply characterized. As expected, multiple *TP53* mutations were discovered by NGS but, when looking at their dynamics rather than mere abundance, only poor responders displayed concomitant increase of TP53 variant allele frequencies (VAFs) and protein expression (Fig. 1e), thus supporting a prognostic role for cancer-related alterations in RMs. These findings were then confirmed in additional samples consecutively collected from patients #2 and #3 (Suppl. Figures 6–7). Again, the impact of p.P72R TP53 SNP on clinical outcome and reduced treatment response was documented (Suppl. Figure 6b-c). Furthermore, to refine our biomarker-based strategy, we integrated a previously validated microRNA signature including miR-21-5p, miR-21-3p, miR-96-5p and miR-429 (see Suppl. Methods), identified as TP53-dependent [8] and with prognostic relevance in HNSCC tissues [9]. When RMs were assessed, a significant decrease of these microRNAs expression was observed in good responders, while an increase was detected for poor ones, correlating with TP53 VAFs (Fig. 2a). Moreover, differential microRNA expression was observed in RMs or pre-cancerous lesions (e.g., pseudoepitheliomatous hyperplasia, PEH) accordingly to their evolution into tumor relapse (Fig. 2b), suggesting a possible role as surrogates for outcome prognostication. However, due to the limitations provided by tissue-based analysis, we decided to move toward liquid biopsy (LB), looking for any non-invasive biomarker(s). No clear association with time to recurrence or clinical outcome was observed for circulating tumor DNAs (ctDNAs) (Suppl. Figure 8a). Conversely, circulating microRNAs included into our signature (miR-21-5p, miR-21-3p ad miR-96-5p, see suppl. methods) appeared upregulated in post-surgery samples as compared to pre-surgery ones only for relapsing cases (Fig. 2c and Suppl. Figure 8b). This was validated also in a second, more heterogenous cohort of 49 HNSCCs (Group 2, Suppl. Figure 1a, Suppl. Tables, sheet 3). Importantly, ROC curve and KM analyses demonstrated that our signature works as an independent diagnostic and prognostic biomarker (Fig. 2d; Suppl. Figure 9), early predicting the risk of local recurrence. Notably, the highest prognostic value was reached 15 days after surgery suggesting microRNAs shedding from residual/hidden tumor cells or pre-malignant lesions (Fig. 2d). Moreover, when combined with RM mutational status, they increased the prognostic and diagnostic significance both at 1 and 15 days after surgery (Fig. 2e-f), suggesting existing links also with RMs. To better clarify this, we finally analyzed expression data obtained from culturing RMs in the presence of Cal27 tumor cells conditioned (CM) or complete media. A prominent higher expression of miR-21-3p and miR-21-5p in RMs of recurrent patients cultured with CM vs. controls was noted when compared to not-recurrent cases (Fig. 2g). Also, since microRNAs shedding may be modulated by tumors, we analyzed their expression in RMs after leaving 72 hours of culture in complete media. Of note, miR-96-5p was highly expressed only in RMs from recurrent patients (Fig. 2h), indicating a differential ability to produce this microRNA between recurrent vs. not-recurrent patients, and further corroborating previous ex-vivo data [10], i.e. post-surgery circulating microRNA levels observed in our cohort (Fig. 2c-d).

Overall, by profiling 69 HNSCC patients, we showed that integration of NGS and dPCR analyses on RMs and blood samples may enable prognostication of patient outcome, ultimately predicting tumor relapse. Particularly, the association of a cost-effective, HNSCC-oriented NGS panel with our 4-microRNA signature offers the opportunity to longitudinally document HNSCC evolution, even in those tissues declared as histologically negative. Given the heterogeneity of our cohort, further studies considering the differences in terms of age, sex and smoking habits, which may impact on clinical outcome, are needed. Moreover, histologically tumor-free tissues still retain a key biological role for cancer development, thus their investigation would be instrumental to increase sensitivity/specificity of HNSCC monitoring and, ultimately, for appropriate post-operative management.

### Electronic supplementary material

Below is the link to the electronic supplementary material.

Supplementary Figure 1. Study design and patient features. (a) Two groups of HNSCC patients (n=69 in total), referred to our Institute for surgical resection of their primitives, were consecutively enrolled between 2013 and 2017. Clinical characteristics of our cohort is detailed in Suppl. Tables, sheets 1-3. Analysis of mutational and/or microRNA profiles were performed on selected cohorts. Numbers of patients included in each of them are indicated together with the specific figures describing molecular results. (b) Patient characteristics of the intersection cases (n=28) between group 1 and 2. RMs: resection margins; pts: patients.

Supplementary Figure 2. Clinical features and molecular profiling of case#1. (a) Clinical history including therapies, sampling and MRI demonstrating tumor extend before surgery of either primary tumor or relapse. (b) Variant allele frequencies of TP53 p.R273H mutation in patient tissues according to NGS and dPCR. Samples related to the diagnosis or recurrence are described in the upper and lower panels, respectively. (c) Immunohistochemistry of TP53 protein expression in tissues from primary tumor, matched recurrence and corresponding resection margins. NED: no evidence of the disease; VAF: variant allele frequency; na: not available; nd: not determined.

Supplementary Figure 3. linical features and molecular profiling of case#2. (a) Clinical history including therapies, sampling and MRI demonstrating tumor extend before surgery of either primary tumor or relapse. (b) Variant allele frequencies of TP53 p.R273H mutation in patient tissues according to NGS and dPCR. Samples related to the diagnosis or recurrence are described in the upper and lower panels, respectively. (c) Immunohistochemistry of TP53 protein expression in tissues from primary tumor, matched recurrence and corresponding resection margins. NED: no evidence of the disease; VAF: variant allele frequency; na: not available; nd: not determined.

Supplementary Figure 4. Clinical features and molecular profiling of case#3. (a) Clinical history including therapies, sampling and MRI demonstrating tumor extend before surgery of either primary tumor or relapse. (b) Variant allele frequencies of TP53 mutations in patient tissues according to NGS and dPCR. Samples related to the diagnosis or recurrence are described in the upper and lower panels, respectively. (c) Immunohistochemistry of TP53 protein expression in tissues from primary tumor, matched recurrence and corresponding resection margins. NED: no evidence of the disease; VAF: variant allele frequency; na: not available; nd: not determined.

Supplementary Figure 5. Clinical features and molecular profiling of case#4. (a) Clinical history including therapies, sampling and MRI demonstrating tumor extend before surgery of either primary tumor or relapse. (b) Variant allele frequencies of TP53 mutations in patient tissues according to NGS and dPCR. Samples related to the diagnosis or recurrence are described in the upper and lower panels, respectively. (c) Immunohistochemistry of TP53 protein expression in tissues from primary tumor and matched recurrence. NED: no evidence of the disease; VAF: variant allele frequency; na: not available; nd: not determined.

Supplementary Figure 6. Mutational profiling and analysis of TP53 p.P72R polymorphism in longitudinal tissue samples. (a) dPCR analysis of TP53 mutations in resection margins of pt#3 (blue bars) or resection margin and PEH of pt#2 (red bars). Cumulative TP53 VAFs calculated by adding all variant allele frequencies of each specific TP53 mutation are shown. (b) 2D plots representing the wild type (P, orange) and mutated allele (R, violet) in primary tumors/lymph node collected at the time of diagnosis and matched recurrences. (c) Histograms of TP53 p.P72R polymorphism percentages according to clinical outcome (blue: poor responders; brown: good responders). VAF: variant allele frequency.

Supplementary Figure 7. IHC analysis of TP53 in consecutive tissue samples. TP53 protein expression in tissues from primary tumors of pt#3 (left) and primary tumor and PEH of pt#2 (right).

Supplementary Figure 8. dPCR and RT-qPCR analysis of ctDNAs and circulating microRNAs. Plasma and sera were collected from HNSCC patients at different time points and assessed for ctDNAs and/or circulating microRNAs expression by either dPCR (mutations) or RT-qPCR (microRNAs). (a) Representative dPCR analysis of baseline blood samples from pts#2 and #3 demonstrating the presence of TP53 ctDNAs (purple dots) into the circulation. Orange, purple, green and black dots depict wild-type, mutated, double-positives and not amplified dPCR spots, respectively. Variant allele frequencies are indicated. (b) Before-after plots showing the modulation of microRNA signature (miR-21-5p, miR-21-3p and miR-96-5p) in serum samples collected before (a) or 1/15 days post-surgery (b-c). Patient #5, who never experienced recurrence, is indicated in blue and has been included as control. Mutational analysis of its tissues shows the presence of TP53 mutation only in tumor tissue (see sample#3 in Suppl. Tables, sheet 4). NTC: no template control; VAF: variant allele frequency.

Supplementary Figure 9. ROC curves of microRNAs expression in liquid biopsy. ROC curve analyses according to microRNAs signature expression at 1 day (left) or 15 days post-surgery (right). The different colors are related to the different microRNAs.

Supplementary Tables

Supplementary Methods

## Data Availability

Raw data and related analyses supporting study findings are available upon reasonable request to the corresponding author (BG).

## Abbreviations

CM: conditioned media
ctDNAs: circulating tumor DNAs
dPCR: digital PCR
HNSCC: head and neck squamous cell carcinoma
HR: hazard ratio
KM: Kaplan-Meier
LB: liquid biopsy
NGS: next generation sequencing
PEH: pseudoepitheliomatous hyperplasia
RFS: recurrence free survival
RMs: resection margins
SNP: single-nucleotide polymorphism
VAF: variant allele frequency

## References

[1] Brouwer de Koning SG, Schaeffers A, Schats W, van den Brekel MWM, Ruers TJM, Karakullukcu MB (2021). Assessment of the deep resection margin during oral cancer surgery: a systematic review. Eur J Surg Oncol.

[2] Marret G, Bièche I, Dupain C, Borcoman E, du Rusquec P, Ricci F (2021). Genomic alterations in Head and Neck squamous cell carcinoma: level of evidence according to ESMO Scale for clinical actionability of molecular targets (ESCAT). JCO Precision Oncol.

[3] Cho J, Johnson DE, Grandis JR (2018). Therapeutic implications of the Genetic Landscape of Head and Neck Cancer. Semin Radiat Oncol.

[4] Comprehensive genomic characterization (2015). Of head and neck squamous cell carcinomas. Nature.

[5] Moe SE, Erland FA, Fromreide S, Lybak S, Brydoy M, Dongre HN et al. The TP53 Codon 72 Arginine Polymorphism Is Found with Increased TP53 Somatic Mutations in HPV(-) and in an Increased Percentage among HPV(+) Norwegian HNSCC Patients. Biomedicines. 2023;11(7). 10.3390/biomedicines11071838 PMC10376802 37509476

[6] Bergamaschi D, Gasco M, Hiller L, Sullivan A, Syed N, Trigiante G (2003). p53 polymorphism influences response in cancer chemotherapy via modulation of p73-dependent apoptosis. Cancer Cell.

[7] Vikhanskaya F, Siddique MM, Kei Lee M, Broggini M, Sabapathy K (2005). Evaluation of the combined effect of p53 codon 72 polymorphism and hotspot mutations in response to anticancer drugs. Clin Cancer Res.

[8] Ganci F, Sacconi A, Bossel Ben-Moshe N, Manciocco V, Sperduti I, Strigari L (2013). Expression of TP53 mutation-associated microRNAs predicts clinical outcome in head and neck squamous cell carcinoma patients. Ann Oncol.

[9] Ganci F, Sacconi A, Manciocco V, Covello R, Benevolo M, Rollo F (2017). Altered peritumoral microRNA expression predicts head and neck cancer patients with a high risk of recurrence. Mod Pathol.

[10] Vahabi M, Pulito C, Sacconi A, Donzelli S, D’Andrea M, Manciocco V (2019). Mir-96-5p targets PTEN expression affecting radio-chemosensitivity of HNSCC cells. J Exp Clin Cancer Res.

