# Correction: Combined TP53 status in tumor-free resection margins and circulating microRNA profiling predicts the risk of locoregional recurrence in head and neck cancer

**DOI:** 10.1186/s40364-024-00582-0

**Published:** 2024-03-26

**Authors:** Federica Ganci, Matteo Allegretti, Carlotta Frascolla, Francesca Spinella, Francesca Rollo, Andrea Sacconi, Valentina De Pascale, Alina Catalina Palcau, Valentina Manciocco, Mariavittoria Vescovo, Ettore Cotroneo, Francesca Blandino, Maria Benevolo, Renato Covello, Paola Muti, Sabrina Strano, Antonello Vidiri, Giulia Fontemaggi, Raul Pellini, Giovanni Blandino

**Affiliations:** 1 Translational Oncologic Research Unit, IRCCS Regina Elena National Cancer Institute, Via Elio Chianesi 53, 00144 Rome, Italy; 2 Department of Research and Development, Eurofins Genoma Group, Rome, Italy; 3 Clinical and Technical Department Management, Eurofins Genoma Group, Rome, Italy; 4 Pathology, IRCCS Regina Elena National Cancer Institute, Via Elio Chianesi 53, 00144 Rome, Italy; 5 Otolaryngology-Head and Neck Surgery, IRCCS Regina Elena National Cancer Institute, Via Elio Chianesi 53, 00144 Rome, Italy; 6 Department of Health Research Methods, Evidence, and Impact, Faculty of Health Sciences, McMaster University, Hamilton, ON Canada; 7 Department of Biomedical, Surgical and Dental Sciences, University of Milan, Milan, Italy; 8 SAFU Unit, IRCCS Regina Elena National Cancer Institute, Via Elio Chianesi 53, 00144 Rome, Italy; 9 Radiology and Diagnostic Imaging, IRCCS Regina Elena National Cancer Institute, Via Elio Chianesi 53, 00144 Rome, Italy

**Correction** **:** **Biomark Res (2024) 12:32**

**https://doi.org/10.1186/s40364-024-00576-y**

The original article [1] mistakenly presented each author's name with Given and Family Names inverted. The authors' names have since been amended.
