# Supplementary material for: Combined TP53 status in tumor-free resection margins and circulating microRNA profiling predicts the risk of locoregional recurrence in head and neck cancer
Source: Biomark Res. 2024 Mar 5;12:32. doi: 10.1186/s40364-024-00576-y (PMC10916059; doi:10.1186/s40364-024-00576-y)
Supplement: Supplementary file 1 — Supplementary Figure 1. Study design and patient features. (a) Two groups of HNSCC patients (n=69 in total), referred to our Institute for surgical resection of their primitives, were consecutively enrolled between 2013 and 2017. Clinical characteristics of our cohort is detailed in Suppl. Tables, sheets 1-3. Analysis of mutational and/or microRNA profiles were performed on selected cohorts. Numbers of patients included in each of them are indicated together with the specific figures describing molecular results. (b) Patient characteristics of the intersection cases (n=28) between group 1 and 2. RMs: resection margins; pts: patients. [file 40364_2024_576_MOESM1_ESM.pptx]

## Slide 1
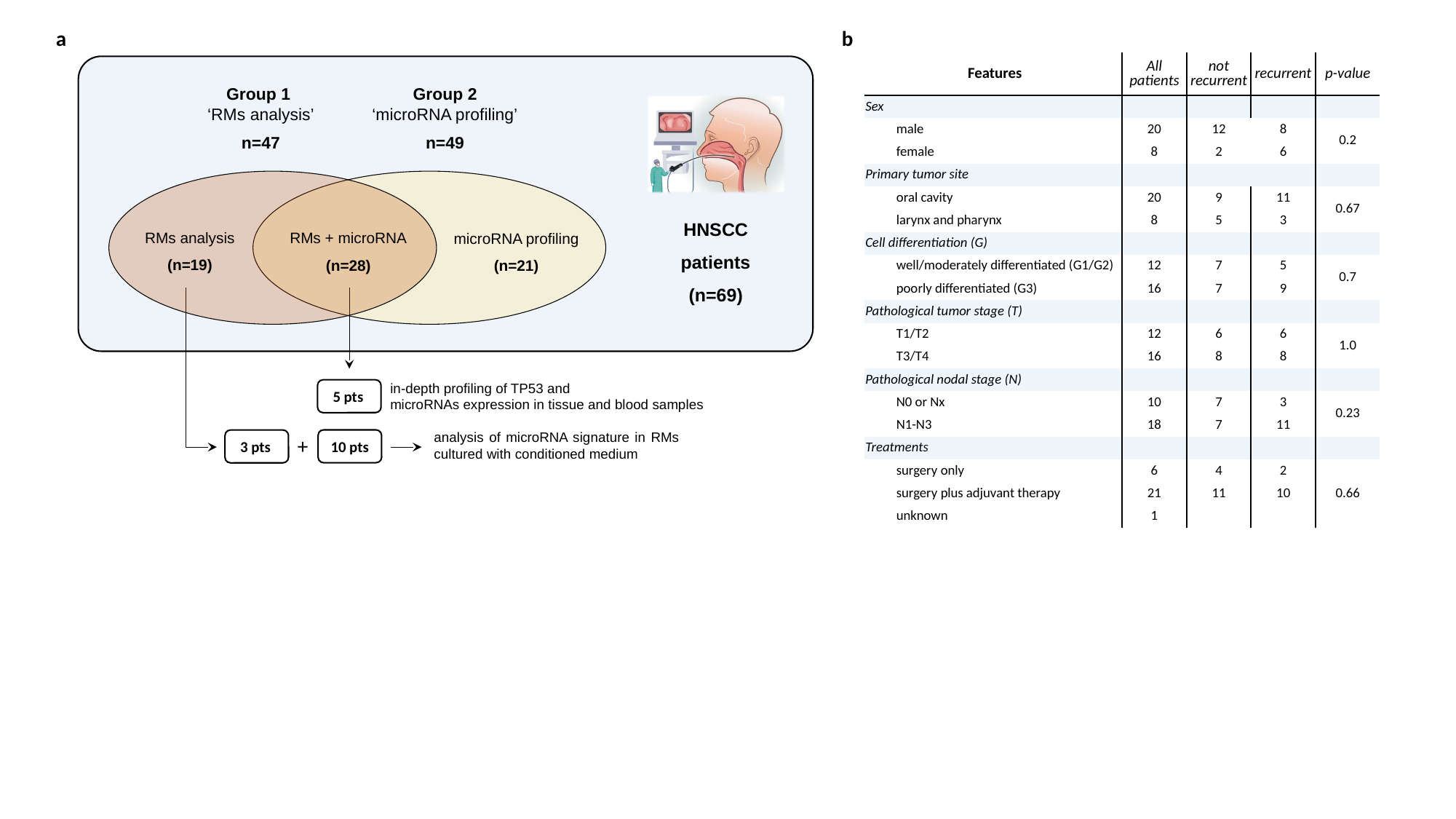

a
b
| Features | All patients | not recurrent | recurrent | p-value |
| --- | --- | --- | --- | --- |
| Sex | | | | |
| male | 20 | 12 | 8 | 0.2 |
| female | 8 | 2 | 6 | |
| Primary tumor site | | | | |
| oral cavity | 20 | 9 | 11 | 0.67 |
| larynx and pharynx | 8 | 5 | 3 | |
| Cell differentiation (G) | | | | |
| well/moderately differentiated (G1/G2) | 12 | 7 | 5 | 0.7 |
| poorly differentiated (G3) | 16 | 7 | 9 | |
| Pathological tumor stage (T) | | | | |
| T1/T2 | 12 | 6 | 6 | 1.0 |
| T3/T4 | 16 | 8 | 8 | |
| Pathological nodal stage (N) | | | | |
| N0 or Nx | 10 | 7 | 3 | 0.23 |
| N1-N3 | 18 | 7 | 11 | |
| Treatments | | | | |
| surgery only | 6 | 4 | 2 | 0.66 |
| surgery plus adjuvant therapy | 21 | 11 | 10 | |
| unknown | 1 | | | |
Group 1
‘RMs analysis’
n=47
Group 2
‘microRNA profiling’
n=49
HNSCC patients
(n=69)
RMs analysis
(n=19)
RMs + microRNA
(n=28)
microRNA profiling
(n=21)
in-depth profiling of TP53 and
microRNAs expression in tissue and blood samples
5 pts
analysis of microRNA signature in RMs cultured with conditioned medium
+
10 pts
3 pts
