# Supplementary material for: Combined TP53 status in tumor-free resection margins and circulating microRNA profiling predicts the risk of locoregional recurrence in head and neck cancer
Source: Biomark Res. 2024 Mar 5;12:32. doi: 10.1186/s40364-024-00576-y (PMC10916059; doi:10.1186/s40364-024-00576-y)
Supplement: Supplementary file 3 — Supplementary Figure 3. linical features and molecular profiling of case#2. (a) Clinical history including therapies, sampling and MRI demonstrating tumor extend before surgery of either primary tumor or relapse. (b) Variant allele frequencies of TP53 p.R273H mutation in patient tissues according to NGS and dPCR. Samples related to the diagnosis or recurrence are described in the upper and lower panels, respectively. (c) Immunohistochemistry of TP53 protein expression in tissues from primary tumor, matched recurrence and corresponding resection margins. NED: no evidence of the disease; VAF: variant allele frequency; na: not available; nd: not determined. [file 40364_2024_576_MOESM3_ESM.pptx]

## Slide 1
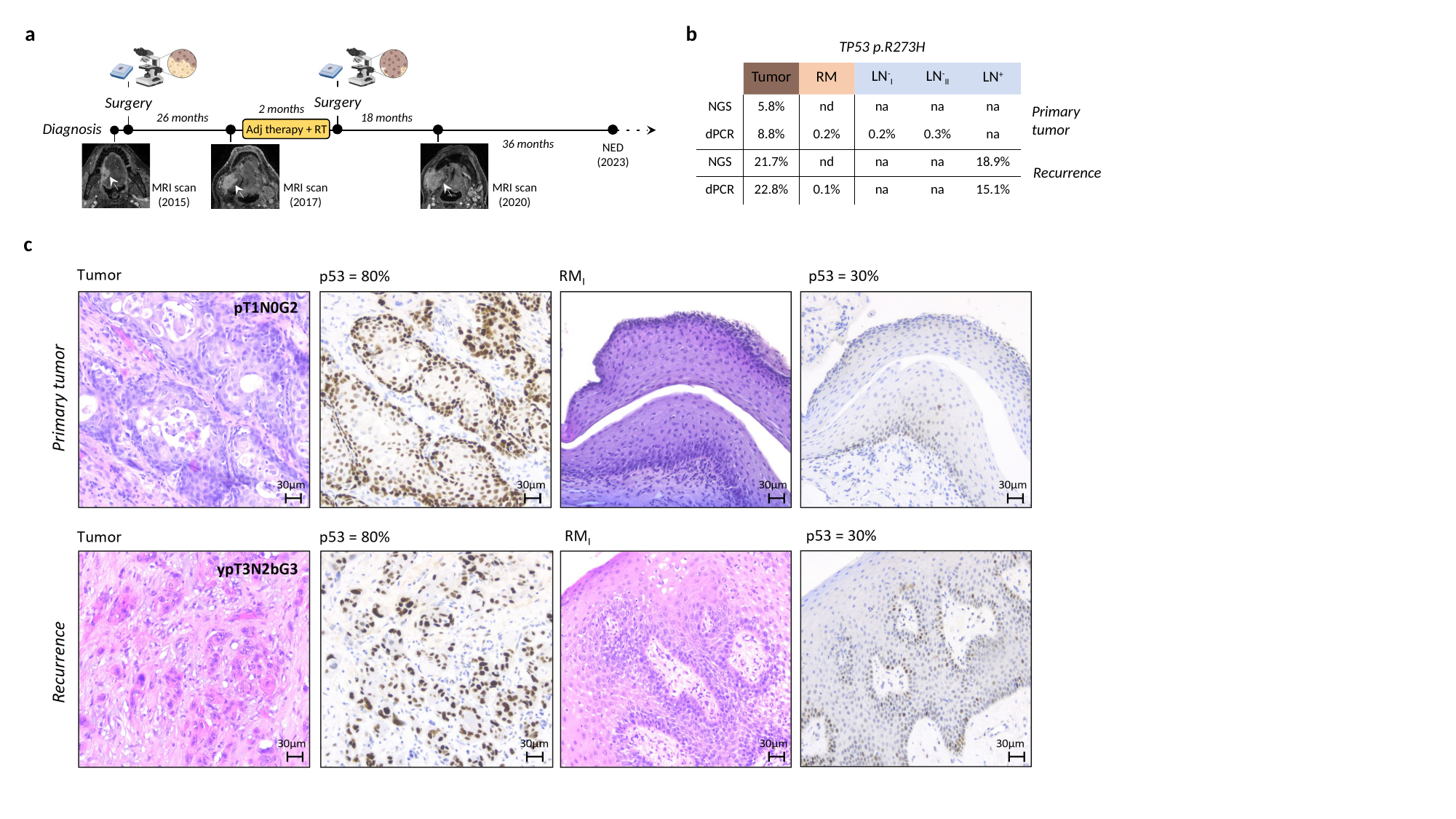

a
b
| | TP53 p.R273H | | | | |
| --- | --- | --- | --- | --- | --- |
| | Tumor | RM | LN-I | LN-II | LN+ |
| NGS | 5.8% | nd | na | na | na |
| dPCR | 8.8% | 0.2% | 0.2% | 0.3% | na |
| NGS | 21.7% | nd | na | na | 18.9% |
| dPCR | 22.8% | 0.1% | na | na | 15.1% |
Surgery
Surgery
2 months
26 months
18 months
Diagnosis
Adj therapy + RT
36 months
NED
(2023)
MRI scan (2015)
MRI scan (2017)
MRI scan (2020)
Primary
tumor
Recurrence
c
