# Supplementary material for: Combined TP53 status in tumor-free resection margins and circulating microRNA profiling predicts the risk of locoregional recurrence in head and neck cancer
Source: Biomark Res. 2024 Mar 5;12:32. doi: 10.1186/s40364-024-00576-y (PMC10916059; doi:10.1186/s40364-024-00576-y)
Supplement: Supplementary file 5 — Supplementary Figure 5. Clinical features and molecular profiling of case#4. (a) Clinical history including therapies, sampling and MRI demonstrating tumor extend before surgery of either primary tumor or relapse. (b) Variant allele frequencies of TP53 mutations in patient tissues according to NGS and dPCR. Samples related to the diagnosis or recurrence are described in the upper and lower panels, respectively. (c) Immunohistochemistry of TP53 protein expression in tissues from primary tumor and matched recurrence. NED: no evidence of the disease; VAF: variant allele frequency; na: not available; nd: not determined. [file 40364_2024_576_MOESM5_ESM.pptx]

## Slide 1
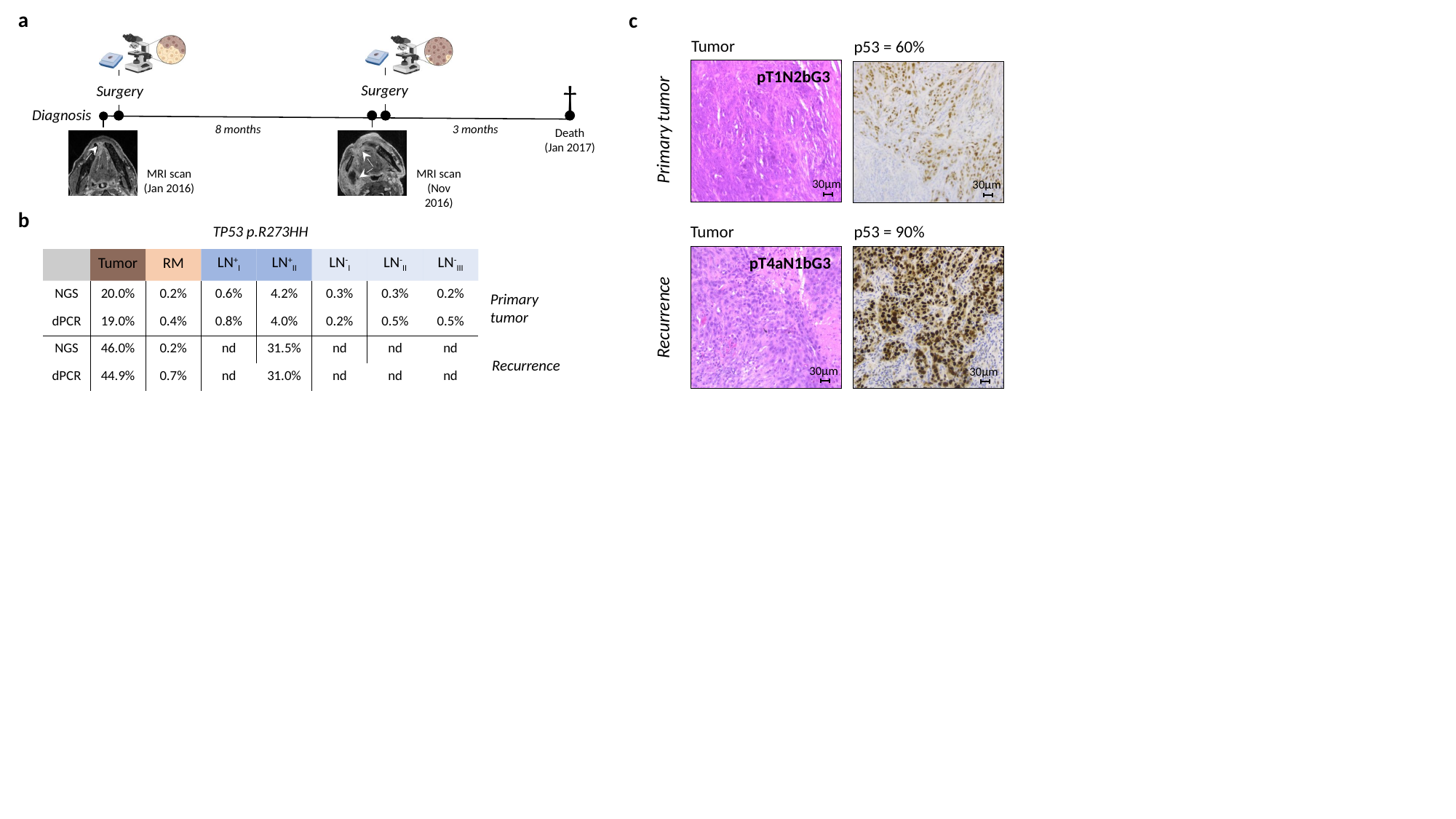

a
c
Tumor
†
Surgery
Surgery
Diagnosis
8 months
3 months
Death
(Jan 2017)
T
N
MRI scan (Jan 2016)
MRI scan (Nov 2016)
p53 = 60%
pT1N2bG3
Primary tumor
30µm
30µm
b
Tumor
p53 = 90%
| TP53 p.R273HH | | | | | | | |
| --- | --- | --- | --- | --- | --- | --- | --- |
| | Tumor | RM | LN+I | LN+II | LN-I | LN-II | LN-III |
| NGS | 20.0% | 0.2% | 0.6% | 4.2% | 0.3% | 0.3% | 0.2% |
| dPCR | 19.0% | 0.4% | 0.8% | 4.0% | 0.2% | 0.5% | 0.5% |
| NGS | 46.0% | 0.2% | nd | 31.5% | nd | nd | nd |
| dPCR | 44.9% | 0.7% | nd | 31.0% | nd | nd | nd |
pT4aN1bG3
Primary
tumor
Recurrence
Recurrence
30µm
30µm
