# Supplementary material for: Combined TP53 status in tumor-free resection margins and circulating microRNA profiling predicts the risk of locoregional recurrence in head and neck cancer
Source: Biomark Res. 2024 Mar 5;12:32. doi: 10.1186/s40364-024-00576-y (PMC10916059; doi:10.1186/s40364-024-00576-y)
Supplement: Supplementary file 6 — Supplementary Figure 6. Mutational profiling and analysis of TP53 p.P72R polymorphism in longitudinal tissue samples. (a) dPCR analysis of TP53 mutations in resection margins of pt#3 (blue bars) or resection margin and PEH of pt#2 (red bars). Cumulative TP53 VAFs calculated by adding all variant allele frequencies of each specific TP53 mutation are shown. (b) 2D plots representing the wild type (P, orange) and mutated allele (R, violet) in primary tumors/lymph node collected at the time of diagnosis and matched recurrences. (c) Histograms of TP53 p.P72R polymorphism percentages according to clinical outcome (blue: poor responders; brown: good responders). VAF: variant allele frequency. [file 40364_2024_576_MOESM6_ESM.pptx]

## Slide 1
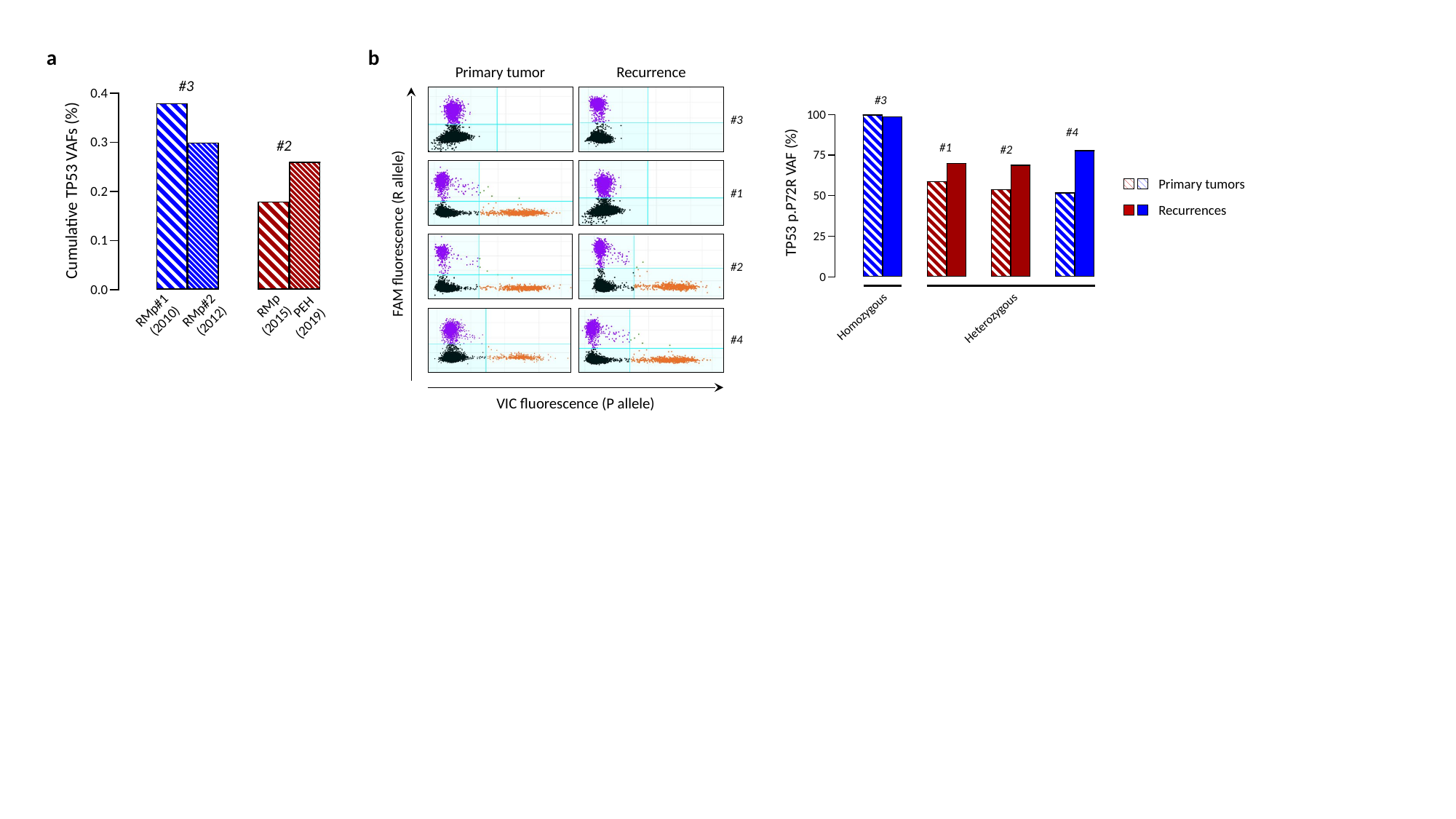

a
b
Primary tumor
Recurrence
#3
#3
#3
#4
#2
#1
#2
Primary tumors
Recurrences
Cumulative TP53 VAFs (%)
TP53 p.P72R VAF (%)
#1
FAM fluorescence (R allele)
#2
RMp#1
(2010)
RMp
(2015)
RMp#2
(2012)
PEH
(2019)
Homozygous
Heterozygous
#4
VIC fluorescence (P allele)
