# Supplementary material for: Combined TP53 status in tumor-free resection margins and circulating microRNA profiling predicts the risk of locoregional recurrence in head and neck cancer
Source: Biomark Res. 2024 Mar 5;12:32. doi: 10.1186/s40364-024-00576-y (PMC10916059; doi:10.1186/s40364-024-00576-y)
Supplement: Supplementary file 7 — Supplementary Figure 7. IHC analysis of TP53 in consecutive tissue samples. TP53 protein expression in tissues from primary tumors of pt#3 (left) and primary tumor and PEH of pt#2 (right). [file 40364_2024_576_MOESM7_ESM.pptx]

## Slide 1
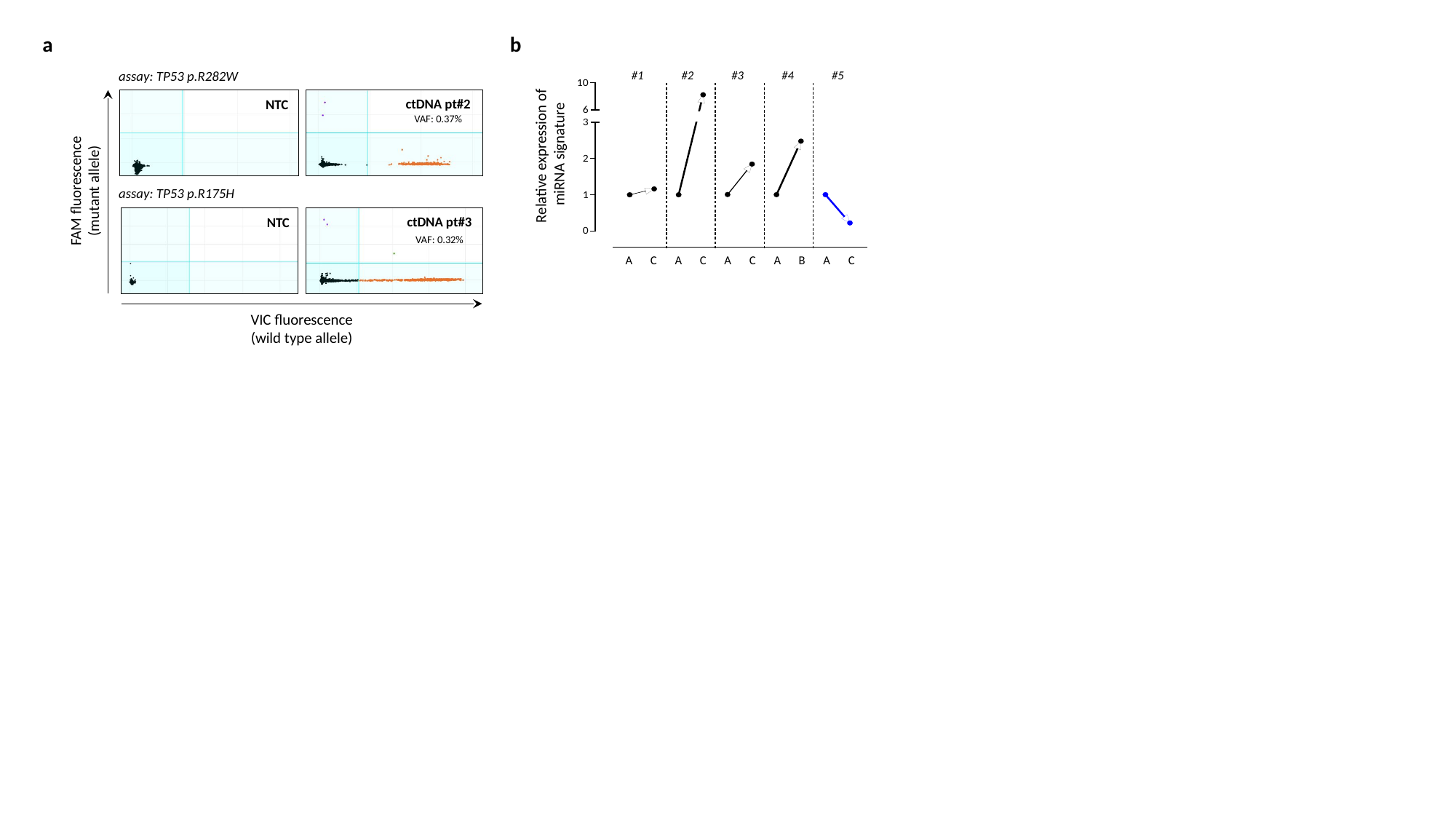

b
a
assay: TP53 p.R282W
#1
#2
#3
#4
#5
ctDNA pt#2
NTC
VAF: 0.37%
Relative expression of
miRNA signature
FAM fluorescence
(mutant allele)
assay: TP53 p.R175H
ctDNA pt#3
NTC
VAF: 0.32%
A
C
A
C
A
C
A
B
A
C
VIC fluorescence
(wild type allele)
