# Supplementary material for: Combined TP53 status in tumor-free resection margins and circulating microRNA profiling predicts the risk of locoregional recurrence in head and neck cancer
Source: Biomark Res. 2024 Mar 5;12:32. doi: 10.1186/s40364-024-00576-y (PMC10916059; doi:10.1186/s40364-024-00576-y)
Supplement: Supplementary file 8 — Supplementary Figure 8. dPCR and RT-qPCR analysis of ctDNAs and circulating microRNAs. Plasma and sera were collected from HNSCC patients at different time points and assessed for ctDNAs and/or circulating microRNAs expression by either dPCR (mutations) or RT-qPCR (microRNAs). (a) Representative dPCR analysis of baseline blood samples from pts#2 and #3 demonstrating the presence of TP53 ctDNAs (purple dots) into the circulation. Orange, purple, green and black dots depict wild-type, mutated, double-positives and not amplified dPCR spots, respectively. Variant allele frequencies are indicated. (b) Before-after plots showing the modulation of microRNA signature (miR-21-5p, miR-21-3p and miR-96-5p) in serum samples collected before (a) or 1/15 days post-surgery (b-c). Patient #5, who never experienced recurrence, is indicated in blue and has been included as control. Mutational analysis of its tissues shows the presence of TP53 mutation only in tumor tissue (see sample#3 in Suppl. Tables, sheet 4). NTC: no template control; VAF: variant allele frequency. [file 40364_2024_576_MOESM8_ESM.pptx]

## Slide 1
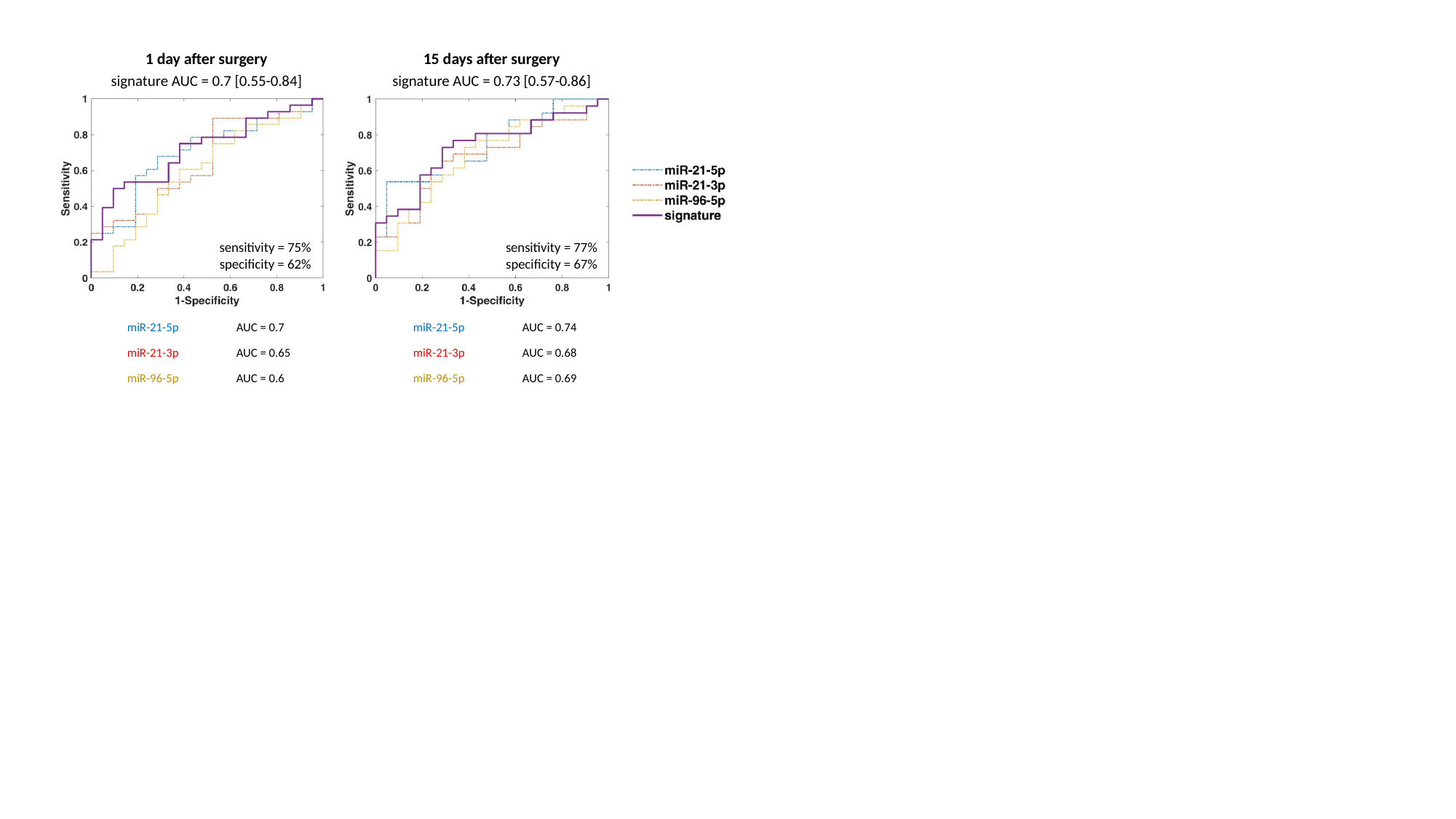

1 day after surgery
15 days after surgery
signature AUC = 0.7 [0.55-0.84]
signature AUC = 0.73 [0.57-0.86]
sensitivity = 77%
specificity = 67%
sensitivity = 75%
specificity = 62%
miR-21-5p 	AUC = 0.7
miR-21-5p 	AUC = 0.74
miR-21-3p 	AUC = 0.65
miR-21-3p 	AUC = 0.68
miR-96-5p 	AUC = 0.6
miR-96-5p 	AUC = 0.69
