# Supplementary material for: Combined TP53 status in tumor-free resection margins and circulating microRNA profiling predicts the risk of locoregional recurrence in head and neck cancer
Source: Biomark Res. 2024 Mar 5;12:32. doi: 10.1186/s40364-024-00576-y (PMC10916059; doi:10.1186/s40364-024-00576-y)
Supplement: Supplementary file 9 — Supplementary Figure 9. ROC curves of microRNAs expression in liquid biopsy. ROC curve analyses according to microRNAs signature expression at 1 day (left) or 15 days post-surgery (right). The different colors are related to the different microRNAs. [file 40364_2024_576_MOESM9_ESM.docx]

**SUPPLEMENTARY METHODS**

*Patient cohort and sample collection*

The present work was carried out at the IRCCS Regina Elena National Cancer Institute in accordance with the Code of Ethics of the World Medical Association (Declaration of Helsinki) for experiments involving humans. Authorization from the Institutional Ethic Review Board (RS868/16) was obtained, and a written informed consent was collected from all human subjects involved in this analysis (n=69). Only patients with HPV-negative, resectable HNSCC of the oral cavity, pharynx or larynx, according to the American Joint Committee on Cancer 8^th^ edition, were enrolled. With respect to sample availability (see below), 2 groups of patients have been defined. A detailed view is presented in Suppl Fig.1.

Formalin-fixed paraffin-embedded (FFPE) blocks of the resected tumors and adjacent RMs, and blood samples, either at the time of first diagnosis or at recurrence (tissues) and during follow up (blood), were retrospectively collected from the Institutional Biobank to profile tissue/circulating mutations or microRNAs. RMs are macroscopically and histologically tumor-free tissues taken at a distance of 1 cm far from the tumor. Sections were initially assessed with immunohistochemistry (IHC) for tumor content by a dedicated pathologist and further used for nucleic acid extraction. Additionally, fresh biopsies from RMs (n=13) of Group 1 patients were collected in 15 ml sterile tubes containing complete media and processed before 2h. Tissues were initially minced and plated into 6-well plates (BD Falcon) for 48h, then 2/4 pieces were moved into a new 6-well plate and cultured for additional 24h in the presence of conditioned media from Cal27 cells (see below).

*Cell lines and retrieval of conditioned media*

Cal27 cell line was obtained from ATCC (Rockville, MD, USA) and grown in RPMI-1640 medium (Invitrogen, Carlsbad, CA, USA) supplemented with 10% fetal bovine serum (FBS), penicillin (200U/ml), streptomycin (200mg/ml), and Amphotericin B antifungal (2.5 µg/ml) (all from Lonza™). Conditioned media were generated from cell cultures (2x10^5^ Cal27 cells) maintained as indicated above for 24h. Media were harvested, centrifuged at 1200x g for 5 min to eliminate traces of detached cells, and clear supernatants were immediately stored in single-use aliquots at −80°C until downstream analysis/use.

*Immunohistochemistry*

Immunohistochemical staining was performed on 3 μm paraffin sections in an automated BOND-III autostainer (Leica Biosystem, Milan, Italy) by a biotin-free polymeric horseradish peroxidase (HRP)-linker antibody conjugate system (Leica Biosystem) using the monoclonal antibody anti-human p53 clone DO-7 (1:100 dilution for 30 minutes, Leica Biosystem) after antigen retrieval by heating in Bond™ Epitope Retrieval Solution 1.

*HPV detection and genotyping*

Briefly, samples were evaluated for 32 HPV types (i.e., HPV 6, 11, 16, 18, 26, 31, 33, 35, 39, 40, 42, 43, 44, 45, 51, 52, 53, 54, 56, 58, 59, 61, 62, 66, 67, 68, 70, 73, 81, 82, 83, 89) by Inno-LiPA HPV Genotyping Extra II (Fujirebio, Rome, Italy). The detection of amplicons was performed by TENDIGO™ (Fujirebio).

*Samples processing and nucleic acids extraction*

Plasma and sera were isolated from whole blood samples within 1 hour from the collection by centrifugation at 3500 rpm for 20 minutes at 4°C, and stored at -80° in single-use aliquots until extraction. DNA and microRNAs were obtained by using the QIAamp DNA FFPE tissue kit (Qiagen, Hilden, Germany) and the MagMax mirVana Total RNA isolation kit (Life Technologies, Carlsbad, CA, USA), respectively, according to manufacturer instructions. Tissue DNAs were quantified by the Qubit Fluorimeter 3.0 and the Qubit dsDNA HS Assay Kit (both from Life Technologies).

*microRNAs expression analysis*

Reverse Transcription and RT-qPCR quantification of microRNAs expression in tissues were performed by TaqMan MicroRNA® Assay Reverse kit and Taqman Assays (both from Life Technologies), respectively, according to the manufacturer's protocol. RNU44 and RNU48 were used as endogenous controls to normalize microRNA expression. Differently, liquid biopsy samples have been assessed by performing the reverse transcription and RT-qPCR with the TaqMan™ Advanced microRNA cDNA Synthesis kit and the appropriate advanced TaqMan microRNA® assay (both from Life Technologies), respectively. The synthetic spike-ins cel-miR-39 and 54-3p were included on sera extraction, acting as normalizers for circulating microRNAs. All reactions were performed in triplicate. miR-429 has not been included in the microRNAs expression signature analysis of liquid biopsy because it wasn’t detected in sera samples.

*Library preparation and NGS analysis*

A total of up to 10 ng DNA per sample was used for target amplification of the full coding sequence of *TP53, FAT1* and *CDKN2A* genes by using a patented custom mutational panel (N.102020000017896/WO2022018777). Library preparation and templating were performed using the Ion AmpliSeq Library Kit Plus and the Ion Chef system (Life Technologies). After sequencing on Ion S5, data were analyzed with the Ion Reporter suite v5.18 (Life Technologies). Tumor-related variants were identified by using the on-board variant caller plugin with customized parameters adapted for low VAFs. Only pathogenic mutations as SNVs, multi-nucleotide variants, small insertions, deletions and indels were analyzed and reported. Limit of detection of each specific call have been assessed by running control samples (e.g., genomic DNA from peripheral blood samples) from healthy volunteers.

*Digital PCR analysis*

Custom primers and probes were designed and ordered from Integrated DNA Technologies (IDT, Coralville, IA, USA). Matched tumoral and resection margins as well as blood samples from each patient were run in the same plate using the QuantStudio Absolute Q Digital PCR System (Life Technologies). Reactions were set up in a final volume of 10 μl including 2 μl of 5x Master Mix, 0.5 nM of each forward and reverse primers, 0.25 nM of MGB probe, 7.5 μl of template, and loaded onto dPCR arrays. Only for tissue analysis input DNA was normalized to 20 ng. Thermal cycling was as follows: 10 min at 96.0°C, 40 cycles at 96.0°C for 5 sec and 30 sec at 60.0°C. Threshold values of FAM and YAK fluorescence, representing either mutated and wild-type alleles, were automatically calculated by the on-board software, manually reviewed, and then used for variant allele quantification. Sensitivity and specificity were assessed for all custom-designed assays using positive and negative control samples (e.g., DNAs obtained from cell models harboring the specific mutations or tissue DNAs previously sequenced by other commercially available NGS panels).

*Statistical analysis*

Impact and relevance of microRNA modulation between different group of samples was assessed by the Wilcoxon test. Categorical variables were analyzed by Fisher’s exact test and *p* values below 0.05 were considered statistically significant. Relapse-free (RFS) was plotted by using the Kaplan-Meier (KM) analysis and log-rank test was used to assess differences between curves. A Cox proportional-hazards regression model was built to assess the hazard ratio (HR) in survival analysis. Patients with high and low signals were defined by considering positive and negative *z*-score values. Average expression of microRNAs signature was used to fit a binomial model. Prediction scores from the classifier were then considered to evaluate both true (sensitivity) and false positive rates (1-specificity) in ROC curves. For the ROC curve analysis a logistic regression model using the fold change of post/pre expression of the miRNA signature as a predictor of the response (relapse/no relapse) has been fitted. Then, we computed the ROC curve using probability estimates from the logistic regression model as scores. The pointwise confidence intervals on the true positive rate (TPR) were obtained by vertical averaging and sampling using bootstrap, with the number of bootstrap replicas set to 1000. The analyses were performed by the MATLAB R2022a software. Relative source code is provided below.

%-----ROC curves-------------
%Fit a logistic regression model. pred are the predictors (miRNA signature FC) and resp the
%response (early\late).
mdl = fitglm(pred,resp,'Distribution','binomial','Link','logit');
% Compute the ROC curve. Use the probability estimates from the logistic regression model as scores.
% Compute the pointwise confidence intervals on the true positive rate (TPR) by vertical averaging (VA) and sampling using bootstrap.
% 'NBoot',1000 sets the number of bootstrap replicas to 1000. perfcurve return X, Y, and T values for all scores, and average the Y values (true positive rate) at all X values (false positive rate) using vertical averaging.
scores = mdl.Fitted.Probability;
% [X,Y,T,AUC aa(1,1:2) ] = perfcurve(resp,scores,'1');
[X,Y,T,AUC(1,1:3) aa(1,1:2)] = perfcurve(resp,scores,'1','NBoot',1000);

figure
plot(X,Y)
ylabel('Sensitivity')
xlabel('1-Specificity')
title(strcat(' 15 Days, signature FC, AUC=',num2str(AUC)))

% ----------------------

%----BOX PLOT ----------
xx=1; %1 FOR 1DAY
% mutN= 0 for WT, 1 for mut
% S=miRNA signature Post\Pre expression;
figure
a=S(find(mutN==0 & time==xx));length(a)
a=[a;NaN(50-length(a),1)];
b=S(find(mutN==1 & time==xx));
length(b)
b=[b;NaN(50-length(b),1)];
boxplot([a b],{'WT','MUT'})
ylabel('Post\Pre expression')
[p h]=ranksum(a,b);
title(strcat('1Day,NORMAL PZ, p=',num2str(p)))
%------------------------------------


%---------------KM plotter

 % MatSurv(TimeVar, EventVar, GroupVar,'param', value, ...) creates a Kaplan-Meier plot,
 % a risk table and calculates a log rank p-value
 %
 % [p] = MatSurv( ... ) returns the log rank p-value
 % [p, fh] = MatSurv( ... ) returns both p-value and figure handle
 % [p, fh, stats] = MatSurv( ... ) returns additions stats from log rank test
 % [p, fh, stats] = MatSurv([], [], []) loads test dataset
mutx=mutN(find(time==2));%mutation (0\1) at 15Days (time=2) or 1Days (time=1)
 survx=prog(find(time==2),:); %Relapse status (days in the first column and status in the second column)
 groupvar=cell(length(mutx),1);groupvar(find(mutx==0))={'WT'};groupvar(find(mutx==1))={'MUT'};
[p, fh, stats] =MatSurv(survx(:,1), survx(:,2), groupvar) ;%creates a Kaplan-Meier plot,
%----------------------------------

**SUPPLEMENTARY DATA**

*Patient clinical histories*

To corroborate the prognostic impact of TP53 mutational status in RMs for early prediction of recurrence, we selected 4 patients from Group 1 which were characterized by different clinical behaviors (e.g., good outcome *vs* poor outcome). First (#1) and second (#2) patients are 62- and 70-year-old women, respectively, never smokers, without a previous history of alcohol abuse. Case #1 was referred to our hospital for a cancer lesion of the oral cavity (ventral surface of the tongue), moderately differentiated (G2), negative for the presence of lymph node metastasis (pT2N0M0) while the second (#2) joint our cancer center for a lesion of the oral cavity (right hemi-lingual), moderately differentiated (G2), negative for the presence of lymph node metastasis (pT1N0M0). The additional third (#3) and fourth (#4) patients were 66- and 81-year-old men, respectively, both heavy smokers. Case #3 was referred to our hospital due to the development of 2 different primary HNSCC tumors (2010 and 2014), located on palatine tonsil (pT1N0G3) and anterior mouth floor (pT1N0G2), respectively. Patient #4 received diagnosis for a lesion in the oral cavity (anterior oral floor), poorly differentiated (G3), and positive for the presence of lymph node metastasis (pT3N2bM0). All these cases were scheduled for tumor resection at our Institute. Biopsy-proved recurrences were treated by surgery alone for case #4, surgery plus chemo-radiotherapy for pt#1 while cases #2 and #3 underwent surgery with only adjuvant radiotherapy due to their age. Case #2 developed two recurrences (Fig. 3a), 2 and 5 years later the onset of the primary tumor, respectively. A fifth case (#5) has been used as control for microRNAs expression analysis in liquid biopsy (Suppl. Fig. 8b). He is a 58 years-old man, never smoker, without a previous history of alcohol abuse, never develop recurrence, distant metastasis or other primitive tumors. He was referred to our hospital for a cancer lesion of the oropharynx (base of tongue), HPV-negative, moderately differentiated (G2), negative for the presence of lymph node metastasis (pT1N0M0) which was treated by surgery alone. His follow up time was of 7 years.

**SUPPLEMENTARY FIGURE LEGENDS**

**Suppl. Fig. 1. Study design and patient features.** (a) Two groups of HNSCC patients (n=69 in total), referred to our Institute for surgical resection of their primitives, were consecutively enrolled between 2013 and 2017. Clinical characteristics of our cohort is detailed in Suppl. Tables, sheets 1-3. Analysis of mutational and/or microRNA profiles were performed on selected cohorts. Numbers of patients included in each of them are indicated together with the specific figures describing molecular results. (b) Patient characteristics of the intersection cases (n=28) between group 1 and 2. RMs: resection margins; pts: patients.

**Suppl. Fig. 2.** **Clinical features and molecular profiling of case#1.** (a) Clinical history including therapies, sampling and MRI demonstrating tumor extend before surgery of either primary tumor or relapse. (b) Variant allele frequencies of TP53 p.R273H mutation in patient tissues according to NGS and dPCR. Samples related to the diagnosis or recurrence are described in the upper and lower panels, respectively. (c) Immunohistochemistry of TP53 protein expression in tissues from primary tumor, matched recurrence and corresponding resection margins. NED: no evidence of the disease; VAF: variant allele frequency; na: not available; nd: not determined.

**Suppl. Fig. 3.** **Clinical features and molecular profiling of case#2.** (a) Clinical history including therapies, sampling and MRI demonstrating tumor extend before surgery of either primary tumor or relapse. (b) Variant allele frequencies of TP53 p.R273H mutation in patient tissues according to NGS and dPCR. Samples related to the diagnosis or recurrence are described in the upper and lower panels, respectively. (c) Immunohistochemistry of TP53 protein expression in tissues from primary tumor, matched recurrence and corresponding resection margins. NED: no evidence of the disease; VAF: variant allele frequency; na: not available; nd: not determined.

**Suppl. Fig. 4. Clinical features and molecular profiling of case#3.** (a) Clinical history including therapies, sampling and MRI demonstrating tumor extend before surgery of either primary tumor or relapse. (b) Variant allele frequencies of TP53 mutations in patient tissues according to NGS and dPCR. Samples related to the diagnosis or recurrence are described in the upper and lower panels, respectively. (c) Immunohistochemistry of TP53 protein expression in tissues from primary tumor, matched recurrence and corresponding resection margins. NED: no evidence of the disease; VAF: variant allele frequency; na: not available; nd: not determined.

**Suppl. Fig. 5. Clinical features and molecular profiling of case#4.** (a) Clinical history including therapies, sampling and MRI demonstrating tumor extend before surgery of either primary tumor or relapse. (b) Variant allele frequencies of TP53 mutations in patient tissues according to NGS and dPCR. Samples related to the diagnosis or recurrence are described in the upper and lower panels, respectively. (c) Immunohistochemistry of TP53 protein expression in tissues from primary tumor and matched recurrence. NED: no evidence of the disease; VAF: variant allele frequency; na: not available; nd: not determined.

**Suppl. Fig. 6. Mutational profiling and analysis of TP53 p.P72R polymorphism in longitudinal tissue samples.** (a) dPCR analysis of TP53 mutations in resection margins of pt#3 (blue bars) or resection margin and PEH of pt#2 (red bars). Cumulative TP53 VAFs calculated by adding all variant allele frequencies of each specific TP53 mutation are shown. (b) 2D plots representing the wild type (P, orange) and mutated allele (R, violet) in primary tumors/lymph node collected at the time of diagnosis and matched recurrences. (c) Histograms of TP53 p.P72R polymorphism percentages according to clinical outcome (blue: poor responders; brown: good responders). VAF: variant allele frequency.

**Suppl. Fig. 7. IHC analysis of TP53 in consecutive tissue samples.** TP53 protein expression in tissues from primary tumors of pt#3 (left) and primary tumor and PEH of pt#2 (right).

**Suppl. Fig 8. dPCR and RT-qPCR analysis of ctDNAs and circulating microRNAs.** Plasma and sera were collected from HNSCC patients at different time points and assessed for ctDNAs and/or circulating microRNAs expression by either dPCR (mutations) or RT-qPCR (microRNAs). (a) Representative dPCR analysis of baseline blood samples from pts#2 and #3 demonstrating the presence of TP53 ctDNAs (purple dots) into the circulation. Orange, purple, green and black dots depict wild-type, mutated, double-positives and not amplified dPCR spots, respectively. Variant allele frequencies are indicated. (b) Before-after plots showing the modulation of microRNA signature (miR-21-5p, miR-21-3p and miR-96-5p) in serum samples collected before (a) or 1/15 days post-surgery (b-c). Patient #5, who never experienced recurrence, is indicated in blue and has been included as control. Mutational analysis of its tissues shows the presence of TP53 mutation only in tumor tissue (see sample#3 in Suppl. Tables, sheet 4). NTC: no template control; VAF: variant allele frequency.

**Suppl. Fig 9. ROC curves of microRNAs expression in liquid biopsy.** ROC curve analyses according to microRNAs signature expression at 1 day (left) or 15 days post-surgery (right). The different colors are related to the different microRNAs.
