# Supplementary material for: Combined TP53 status in tumor-free resection margins and circulating microRNA profiling predicts the risk of locoregional recurrence in head and neck cancer
Source: Biomark Res. 2024 Mar 5;12:32. doi: 10.1186/s40364-024-00576-y (PMC10916059; doi:10.1186/s40364-024-00576-y)
Supplement: Supplementary file 11 — Supplementary Methods [file 40364_2024_576_MOESM11_ESM.pptx]

## Slide 1
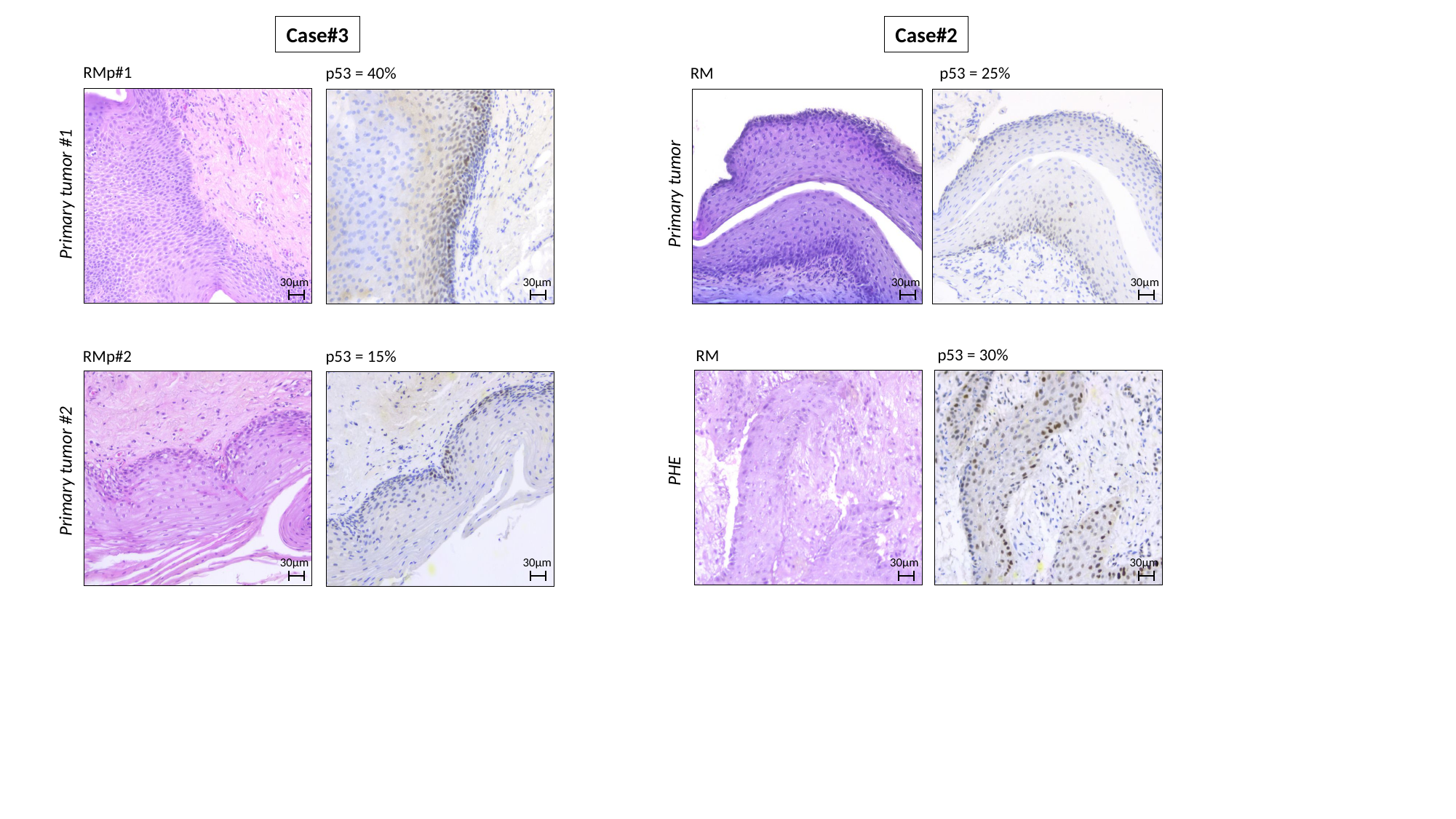

Case#3
Case#2
RMp#1
RM
p53 = 25%
p53 = 40%
Primary tumor #1
Primary tumor
30µm
30µm
30µm
30µm
p53 = 30%
RM
RMp#2
p53 = 15%
Primary tumor #2
PHE
30µm
30µm
30µm
30µm
